# Cellular Proteo-Transcriptomic Changes in the Immediate Early-Phase of Lentiviral Transduction

**DOI:** 10.3390/microorganisms9112207

**Published:** 2021-10-23

**Authors:** Tamás Richárd Linkner, Viktor Ambrus, Balázs Kunkli, Zsófia Ilona Szojka, Gergő Kalló, Éva Csősz, Ajneesh Kumar, Miklós Emri, József Tőzsér, Mohamed Mahdi

**Affiliations:** 1 Laboratory of Retroviral Biochemistry, Department of Biochemistry and Molecular Biology, Faculty of Medicine, University of Debrecen, 4032 Debrecen, Hungary; linkner.tamas@science.unideb.hu (T.R.L.); ambrus.viktor@med.unideb.hu (V.A.); kunkli.balazs@med.unideb.hu (B.K.); 2 Doctoral School of Molecular Cell and Immune Biology, University of Debrecen, 4032 Debrecen, Hungary; kumar.ajneesh@med.unideb.hu; 3 Division of Medical Microbiology, Department of Laboratory Medicine, Lund University, 221 00 Lund, Sweden; zsofia_ilona.szojka@med.lu.se; 4 Proteomics Core Facility, Department of Biochemistry and Molecular Biology, Faculty of Medicine, University of Debrecen, 4032 Debrecen, Hungary; kallo.gergo@med.unideb.hu (G.K.); cseva@med.unideb.hu (É.C.); 5 Department of Medical Imaging, Division of Nuclear Medicine and Translational Imaging, Faculty of Medicine, University of Debrecen, 4032 Debrecen, Hungary; emri.miklos@med.unideb.hu

**Keywords:** HIV-1, HIV-2, lentiviral vectors, transcriptome, proteome, host response

## Abstract

Lentivirus-based vectors derived from human immunodeficiency viruses type 1 and 2 (HIV-1 and 2) are widely used tools in research and may also be utilized in clinical settings. Like their parental virions, they are known to depend on the cellular machinery for successful gene delivery and integration. While most of the studies on cellular proteomic and transcriptomic changes have focused on the late phase of the transduction, studies of those changes in early time-points, especially in the case of HIV-2 based vectors, are widely lacking. Using second generation HIV-1 and 2 vesicular stomatitis virus G protein (VSV-G) pseudotyped lentiviral vectors, we transduced HEK-293T human embryonic kidney cells and carried out transcriptomic profiling at 0 and 2 h time points, with accompanying proteomic analysis at 2 h following transduction. Significant variations were observed in gene expression profile between HIV-1 and HIV-2 transduced samples. Thrombospondin 1 (THBS1), collagens (COL1A2, COL3A1), and eukaryotic translation factors (EIF3CL) in addition to various genes coding for long non-coding RNA (lncRNA) were significantly upregulated 2 h after HIV-2 transduction compared to HIV-1. Label-free quantification mass spectrometry (MS) indicated that seven proteins involved in RNA binding, mRNA transport, and chaperoning were significantly downregulated. The identification of cellular protein targets of lentiviral vectors and their effect on the cellular transcriptome will undoubtedly shed more light on their complex life cycle and may be utilized against infection by their parental lentiviruses. Furthermore, characterizing the early phase of HIV-2 infection may aid in the understanding of its pathomechanism and long incubation period.

## 1. Introduction

The ability of lentiviruses to infect both dividing and non-dividing cells, and to integrate into the host cell’s genome, hence resulting in long-term and stable gene expression, have made them attractive means for the modification of eukaryotic cells. Therefore, lentiviral vectors have been extensively used in clinical research and gene therapy [1]. Additionally, direct in vivo delivery of genes carried by equine infectious anemia virus (EIAV)-based vectors into target tissues has been utilized successfully in a dog model of hemophilia B and patients with advanced neovascular age-related macular degeneration (NVAMD) with evidence of therapeutic potential and a promising safety profile [2,3].

However, there are many issues surrounding the use of lentiviral-based vectors in a clinical setting, such as the risk of insertional mutagenesis, resulting in a subsequent malignant transformation in the patient, and the potential for recombination events leading to the creation of replication-competent lentiviruses, especially during the production phase [4]. 

The human immunodeficiency viruses type 1 and 2 (HIV-1 and HIV-2) belong to the *lentivirinae* class of the *retroviridae* family. They form lipid-enveloped particles around 100 nm in diameter containing a positive-sense single stranded RNA genome [5]. Members of the *retroviridae* family share a similar life cycle. In brief, in the early-phase of infection, after attachment and entry into target cells through either direct membrane fusion or receptor-mediated endocytosis (mediated by the envelope protein and the target receptor), some viral proteins dissociate from the viral core in a process termed uncoating. The viral encoded reverse transcriptase (RT) then converts viral RNA to proviral double-stranded DNA, which then complexes with viral proteins, resulting in the formation of the pre-integration complex (PIC). The formation of PIC is crucial for the nuclear import of the proviral DNA, as the viral protein components of the PIC, such as the matrix protein, integrase, and viral protein R (Vpr), possess nuclear localization signals required for guidance into the nucleus [6,7]. 

Finally, the viral integrase (IN) mediates the integration of the proviral DNA into the host cell’s genome, a process that was found to be heavily influenced by host proteins, such as lens epithelium-derived growth factor (LEDGF/p75) and bromodomain containing proteins (BET), in the case of HIV-1 and gammaretroviruses, respectively [8,9].

In regard to HIV-1, many studies have analyzed interactions between the virus and host factors using genome-wide RNA analysis and proteomic assays [10,11,12,13]. However, data concerning virus–host interactions in the context of HIV-2 are widely lacking, resulting in a lower number of HIV-2-human protein–protein interactions (PPI) in the public databases as compared to HIV-1 [14]. PPIs provide valuable data. They are used to create network-based models of HIV infection, thereby aiding the understanding of the pathomechanism of infection, and may identify targets that can be used to fight the infection [15].

Given all of the above, it is indeed important to characterize the cellular transcriptomic and proteomic changes in the early phase of lentiviral transduction. Identifying early targets of HIV will undoubtedly enrich PPI networks, shedding more light on the pathomechanism of infection, and may help to improve the safety and efficacy of lentiviral-based vectors. Moreover, given the lack of data on HIV-2, we carried out a comparative analysis of cellular proteo-transcriptomic changes upon HIV-1 and 2 pseudovirus transduction in the first 2 h, in order to detect whether or not any difference is present between the two viruses, keeping in mind that HIV-2 has noticeably different replication dynamics and a divergent clinical course of infection [16,17].

## 2. Materials and Methods

### 2.1. Plasmids and Vectors

We utilized second generation lentiviral vectors for HIV-1 and HIV-2 pseudovirion and ‘mock’ production. For HIV-1, the following plasmids were used: psPAX_2,_ as a packaging plasmid, a kindly gift from Dr. D. Trono (University of Geneva Medical School, Geneva, Switzerland); pMD.G, encoding for G glycoprotein of vesicular stomatitis virus (VSV-G); and pWOX-CMV-GFP, as a transfer vector, which was modified to code for mCherry instead of GFP (pWOX-CMV-mCherry) [18]. The following plasmids were used for HIV-2 pseudovirion production: HIV-2 CGP vector; a ROD based HIV-2 protein expression vector which encodes all HIV-2 genes except *nef* and *env*; HIV-2-CRU5SIN-CGW vector; a minimal HIV-2 plasmid containing a GFP expression cassette under a CMV promoter; and pMD.G vector. 

For mock virion production, pTY-EFeGFP vector, a lentiviral transducing vector containing a GFP expression cassette under an EF1α promoter, and pMD.G vectors were used [19].

HIV-2 CGP and HIV-2-CRU5SIN-CGW were a kind gift from Joseph P. Dougherty at the Robert Wood Johnson Medical School (New Brunswick, NJ, USA) [20,21].

### 2.2. Production of HIV-1, HIV-2, and Mock Pseudovirions

HEK-293T human embryonic kidney cells (Invitrogen, CA, USA) were used for the production of pseudovirions. To produce HIV-1 pseudovirions, psPAX_2_, pWOX-CMV-mCherry, and pMD.G plasmids were used in a 3:2:1 ratio. For the production of HIV-2 pseudovirions, HIV-2 CGP, HIV-2-CRU5SIN-CGW, and pMD.G plasmids were used in a ratio of 1:1:1 [21]. To produce mock virions, pTY-EFeGFP and pMD.G vectors were used in 1:1 ratio. Plasmid ratios used were optimal for pseudovirion production.

A day before transfection, HEK-293T cells were passaged in order to achieve 70% confluence (5–6 × 10^6^ cells/mL) on the next day. For HIV-1, a total of 36 μg plasmid DNA, 30 μg for HIV-2, and 20 μg for mock were used for transfection, using polyethylenimine (PEI) (Sigma-Aldrich, St. Louis, MO, USA). Cells were then incubated at 37 °C with 5% CO_2_ for 5–6 h in 5 mL medium containing 1% FBS without antibiotics. The medium was then replaced with 15 mL DMEM containing 10% FBS, 1% penicillin-streptomycin, and 1% glutamine. Supernatant was collected and filtered through a 0.45 μm polyvinylidene fluoride filter (Merck Millipore, Darmstadt, Germany) after 24, 48, and 72 h of transfection, and then pooled together and concentrated by ultracentrifugation (100,000× *g* for 2 h at 4 °C). The pellet containing viral particles was then dissolved in 200 µL phosphate-buffered saline (PBS) and stored at −70 °C. An enzyme-linked immunosorbent assay (ELISA)-based colorimetric reverse transcriptase (RT) assay (Roche Applied Science, Mannheim, Germany) was then used to detect the amount of RT in the virus (HIV-1 and HIV-2) samples according to the manufacturer’s instructions. Quantification of mock pseudovirions was carried out by transduction experiments on HEK-293T cells and measuring the transduction units/mL (TU/mL). The amount was then adjusted to the RT equivalence of HIV-1 and 2 pseudovirions in order to use an equal quantity for transduction experiments.

### 2.3. Transduction of HEK-293T Cells for Transcriptomic Analysis

The day before transduction, HEK-293T cells were plated in a 6-well plate (500,000 cells/well) in 1500 µL of DMEM supplemented with 10% FBS, 1% glutamine, and 1% penicillin-streptomycin. The following day, the medium was discarded and the cells were transduced with 5 ng RT-equivalent of HIV-1/HIV-2 or mock pseudovirions in 1500 µL serum- and antibiotic-free media, complemented with 8 µg/mL polybrene (Sigma, St. Louis, MO, USA). Cells were collected at 0–2 h after transduction, media was discarded, and cells were washed with 500 µL PBS, then suspended in 500 µL TRIzol Reagent (UD-GenoMed Medical Genomic Technologies, Debrecen, Hungary). RNA isolation was carried out according to the protocol from Thermo Fisher Scientific. RNA quality was determined using an Agilent RNA 6000 Nano kit on an Agilent 2100 Bioanalyzer (Agilent Technologies, Waldbronn, Germany). Thereafter, high-throughput sequencing was performed on the MGI DNBSEQ G400 sequencer using MGIEasy RNA Library Prep Set at the Genomic Medicine and Bioinformatics Core Facility of the University of Debrecen.

### 2.4. Transduction of HEK-293T Cells for Proteomic Analysis

The day before transduction, HEK-293T cells were seeded in a T-25 flask (7 × 10^5^) in 5 mL of DMEM supplemented with 10% FBS, 1% glutamine, and 1% penicillin-streptomycin. The following day, the medium was discarded and the cells were transduced with 15 ng RT-equivalent of HIV-1/HIV-2, or mock pseudovirions, in 1500 µL serum- and antibiotic-free media, complemented with 8 µg/mL polybrene (Sigma, St. Louis, MO, USA). The cells were thereafter incubated at 37 °C, 5% CO_2_ for 2 h. The media was discarded, and cells were mechanically detached into 5 mL PBS. After brief centrifugation (6 min, 152× *g*), the pellet was then washed again with 5 mL PBS and finally with 1 mL PBS. After a final centrifugation step, the pellet was stored at −20 °C. 

### 2.5. Transcriptomic Data Analysis

Adapter sequence removal and quality filtering were performed with Trimmomatic v0.36 [22]. Sequencing reads were aligned in HISAT2 v.2.1.0 [23] against the GRCh38 Human Genome Assembly. The reference genome index files were built with Bowtie v.1.2.2 [24]. FeatureCounts v1.6.2 [25] was used to count the total number of reads overlapping each gene, in the default settings using the Ensembl Build GRCh38.101 gtf file [26]. Read counts per gene were normalized using the median-of-ratios method introduced with the DESeq2 tool [27]. Principal component analysis (PCA) of the normalized read counts was used to explore the gene expression profiles for samples over time.

To improve the fold estimates of the expression levels, we have applied shrinkage on the log_2_ fold change results with the lfcShrink function of DESeq2. Genes with an adjusted *p*-value lower than 0.05 and absolute log_2_ fold changes over 0.58 were considered as differentially expressed.

Gene Ontology (GO) enrichment analysis of differentially expressed genes was performed with the ClusterProfiler package [28].

### 2.6. GeLC-MS/MS Analysis

Lysis of the mock, HIV-1, or HIV-2 transduced cells was performed in 100 µL lysis buffer (50 mM Tris pH 8.3, 1 mM EDTA, 17 mM β-mercaptoethanol, 0.5% (V/V) Triton-X100) by three freeze–thaw cycles. The protein concentration of the samples was determined by the Bradford method, and 100 µg of protein in each case was subjected to in-gel digestion followed by liquid chromatography-tandem mass spectrometry (GeLC-MS/MS) analysis [29]. Briefly, samples were run into a 5% SDS-polyacrylamide gel using a 100V current for 20 min. The proteins were stained with PageBlue Protein Staining solution (Thermo Scientific, Waltham, MA, USA), and the stained gel slice was excised, separated to three equal portions, and subjected to in-gel trypsin digestion. Reduction was performed with 20 mM dithiothreitol (Bio-Rad, Hercules, CA, USA) for 1 h at 56 °C, followed by alkylation with 55 mM iodoacetamide (Bio-Rad, Hercules, CA, USA) for 45 min at room temperature in the dark. Overnight trypsin digestion was performed at 37 °C using stabilized MS grade TPCK-treated bovine trypsin (ABSciex, Framingham, MA, USA). The digested peptides were extracted and dried in a speed-vac (Thermo Scientific, Waltham, MA, USA). The peptides were re-dissolved in 33 μL 1% formic acid (VWR Ltd., Radnor, PA, USA) before LC-MS/MS analysis. The peptide concentration of the samples was determined using the BCA method. Prior to mass spectrometry analyses, the samples were spiked with equal amounts of indexed retention time (iRT) peptide mixtures (Biognosys, Schlieren, Switzerland), and the samples were analyzed in duplicates.

Prior to the mass spectrometric analysis, peptides were separated in a 180 min water/acetonitrile gradient using an Easy nLC 1200 nano UPLC (Thermo Scientific, Waltham, MA, USA). The peptide mixtures were desalted in an ACQUITY UPLC Symmetry C18 trap column (20mm × 180 µm, 5 μm particle size, 100 Å pore size, Waters, Milford, MA, USA), followed by separation in Acclaim PepMap RSLC C18 analytical columns (150 mm × 50 μm 2 μm particle size, 100 Å pore size, Thermo Scientific, Waltham, MA, USA). Chromatographic separation was performed using a gradient of 5–7% solvent B over 5 min, followed by a rise to 15% of solvent B over 50 min, and then to 35% solvent B over 60 min. Thereafter, solvent B was increased to 40% over 28 min and then to 85% over 5 min, followed by a 10 min rise to 85% of solvent B, after which the system returned to 5% solvent B in 1 min for a 16 min hold-on. Solvent A was 0.1% formic acid in LC water (Sigma, St. Louis, MO, USA); solvent B was 95% acetonitrile (Sigma, St. Louis, MO, USA) containing 0.1% formic acid. The flow rate was set to 300 nL/min.

Data-dependent analyses were carried out on an Orbitrap Fusion mass spectrometer (Thermo Scientific, Waltham, MA, USA). The 14 most abundant multiply charged positive ions were selected from each survey MS scan using a scan range of 350–1600 *m*/*z* for MS/MS analyses (Orbitrap analyser resolution: 60,000, AGC target: 4.0e5, acquired in profile mode). Collision induced dissociation (CID) fragmentation was performed in the linear ion trap with 35% normalized collision energy (AGC target: 2.0e3, acquired in centroid mode). Dynamic exclusion was enabled during the cycles (exclusion time: 45 s).

### 2.7. Data Analysis of Mass Spectrometry

The acquired LC-MS/MS data were used for protein identification with the help of MaxQuant 1.6.2.10 software [30] searching against the Human SwissProt database (release: 2020.02, 20394 sequence entries), the HIV-1 and HIV-2 SwissProt databases (release: 2020.02, 381 sequence entries for HIV-1 and 109 sequence entries for HIV-2), and against the contaminants database provided by the MaxQuant software. Cys carbamidomethylation, Met oxidation, and N-terminal acetylation were set as variable modifications. A maximum of 2 missed cleavage sites were allowed. Results were imported into Scaffold 4.8.9 software (ProteomeSoftware Inc., Portland, OR, USA). Proteins were accepted with at least 3 identified peptides using 1% protein false discovery rate (FDR) and 0.1% peptide FDR. For label-free quantification, the normalized total precursor intensities were used, and quantitative values of the identified proteins were normalized to the quantitative values of the iRT mixture. The data were also normalized to the concentration of the peptides in the samples determined by BCA method after the digestion.

A mass of mixed effects of ANOVA models were applied, one for each protein, to select the significantly different protein quantities between the investigated groups. Sample and measurement repetitions were modelled as random effects, and the transduced cell groups were modelled as fixed effects [31]. After the linear model fitting, post-hoc tests were applied to determine the *p*-values of group differences, and significant results with an FDR < 0.05 criteria were retained.

## 3. Results

### 3.1. Analysis of Transcriptomic Changes in the First 2 h of HIV-1 and 2 Transduction

To analyse changes in the host transcriptome as a result of transduction by HIV-1 and -2 based lentiviral vectors, RNA-seq analysis was carried out. RNA-seq yielded on average 23 million raw sequencing reads per sample. After read trimming and quality filtering, we obtained an average of 22 million aligned reads per sample, including uniquely aligned reads and multimapper reads. In each sample, over 86% of the reads were uniquely mapped to the ENSEMBL GRCh38.101 human reference sequence (Table S1). Compared to mock-transduced cells, HIV-1 significantly altered the regulation pattern of 4551 genes, out of which 2366 were upregulated. Of the upregulated genes, 1916 coded for proteins, and 98 for products of pseudogenes, while 334 were non-coding RNAs. Moreover, 18 gene products were described in databases as uncategorized genes. On the other hand, transcripts of 2185 genes were decreased by HIV-1, of which 1634 were protein coding, 153 were pseudogenes, 363 were non-coding RNA transcripts, 18 coding for mitochondrial RNA, one for ribozyme, and 16 were products of uncategorized genes (Table S2).

Analysis of the HIV-2 transduced cells revealed RNA of 3422 genes that were differentially altered compared to the mock control. Moreover, 2114 transcripts were found to be upregulated, of which 1716 were protein-coding and 128 were products of pseudogenes, 261 were non-coding RNAs, and nine were products of uncategorized genes. HIV-2 also resulted in the decrease of RNA of 1308 genes, 875 of which were protein-coding, 306 non-coding RNAs, and 16 were coding for mitochondrial RNAs. We also identified products of 83 pseudogenes, one ribozyme coding transcript, and 27 uncategorized RNAs (Table S3).

#### 3.1.1. Differentially Expressed Genes at 0 h Post-Transduction

**Figure 1:**
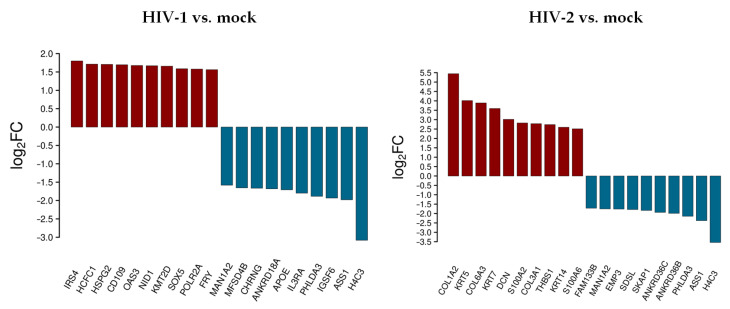
Top 10 selected up- and downregulated genes at the 0 h time-point following transduction. Log_2_ fold change (log_2_FC) of the up- and downregulated transcripts coding for proteins in HIV-1 and HIV-2 transduced HEK-293T cells, compared to mock transduction.

The top 10 differentially up- and downregulated protein-coding genes as a result of HIV-1 and HIV-2 transduction, compared to mock transduction, were selected and visualized, as shown in Figure 1.

At 0 h, HIV-1 resulted in the up-regulation of insulin receptor substrate 4 (IRS4), host cell factor C1 (HCFC1), heparan sulfate proteoglycan 2 (HSPG2), CD109 molecule, 2’-5’-oligoadenylate synthetase 3 (OAS3), nidogen 1 (NID1), lysine methyltransferase 2D (KMT2D), SRY-box transcription factor 5 (SOX5), RNA polymerase II subunit A (POLR2A), and FRY microtubule binding protein (FRY). Immunoglobulin superfamily member 6 (IGSF6), interleukin 3 receptor subunit alpha (IL3RA), apolipoprotein E (APOE), ankyrin repeat domain 18A (ANKRD18A), cholinergic receptor nicotinic gamma subunit (CHRNG), and major facilitator superfamily domain containing 4B (MFSD4B) were downregulated.

HIV-2, on the other hand, resulted in the up-regulation of collagen type I alpha 2 chain (COL1A2), keratin 5 (KRT5), collagen type VI alpha 3 chain (COL6A3), decorin (DCN), S100 calcium binding protein A2 (S100A2), collagen type III alpha 1 chain (COL3A1), thrombospondin 1 (THBS1), keratin 14 (KRT14), S100 calcium binding protein A6 (S100A6), and the down-regulation of ankyrin repeat domain 36B and 36C (ANKRD36B/36C), src kinase associated phosphoprotein 1 (SKAP1), serine dehydratase like (SDSL), epithelial membrane protein 3 (EMP3), and family with sequence similarity 133 member B (FAM133B).

H4 clustered histone 3 (H4C3), argininosuccinate synthase 1 (ASS1), pleckstrin homology like domain family A member 3 (PHLDA3), and mannosidase alpha class 1A member 2 (MAN1A2) were downregulated in both HIV-1 and -2 transduced samples.

A comprehensive list of differentially expressed transcripts can be found in Table S4.

#### 3.1.2. Differentially Expressed Genes at 2 h Post-Transduction

**Figure 2:**
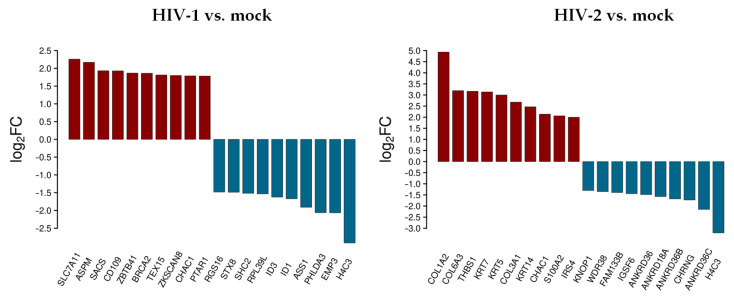
Top 10 selected up- and downregulated protein coding genes 2 h after transduction. log_2_FC of the up- and downregulated transcripts coding for proteins in HIV-1 and HIV-2 transduced HEK-293T cells, respectively, compared to mock transduction.

The top 10 differentially up- and downregulated protein-coding genes as a result of HIV-1 and HIV-2 transduction, respectively, compared to mock transduction were selected, as presented in Figure 2.

In decreasing order of magnitude, HIV-1 resulted in the up-regulation of solute carrier family 7 member 11 (SLC7A11), assembly factor for spindle microtubules (ASPM), sacsin molecular chaperone (SACS), CD109 molecule, zinc finger and BTB domain containing 41 (ZBTB41), BRCA2 DNA repair associated (BRCA2), testis expressed 15, meiosis and synapsis associated (TEX15), zinc finger with KRAB and SCAN domains 8 (ZKSCAN8), and protein prenyltransferase alpha subunit repeat containing 1 (PTAR1), respectively. On the other hand, epithelial membrane protein 3 (EMP3), pleckstrin homology like domain family A member 3 (PHLDA3), argininosuccinate synthase 1 (ASS1), inhibitor of DNA binding 1, and 3 HLH protein (ID1/3), ribosomal protein L39 like (RPL39L), SHC adaptor protein 2 (SHC2), syntaxin 8 (STX8), and regulator of G protein signaling 16 (RGS16) were downregulated.

**Figure 3:**
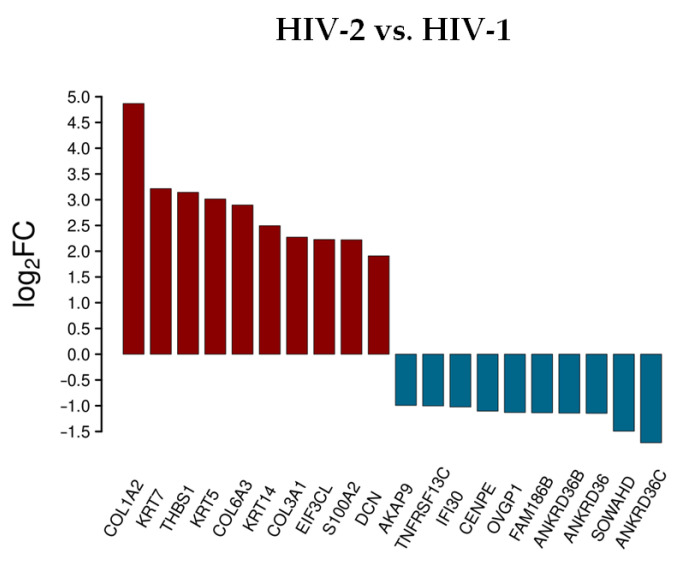
Differential expression of genes post HIV-2 transduction, compared to HIV-1. log_2_FC of the most up and downregulated protein genes detected from HIV-2 transduced cells.

In cells transduced by HIV-2, collagen type I alpha 2 chain (COL1A2), collagen type VI alpha 3 chain (COL6A3), thrombospondin 1 (THBS1), keratin 7 and 5 (KRT7/5), collagen type III alpha 1 chain (COL3A1), keratin 14 (KRT14), S100 calcium binding protein A2 (S100A2), and insulin receptor substrate 4 (IRS4) were upregulated, while ankyrin repeat domain 36C (ANKRD36C), cholinergic receptor nicotinic gamma subunit (CHRNG), ankyrin repeat domains 36B, 18A, 36 (ANKRD36B/18A/36), immunoglobulin superfamily member 6 (IGSF6), family with sequence similarity 133 member B (FAM133B), WD repeat domain 38 (WDR38), and lysine rich nucleolar protein 1 (KNOP1) were downregulated. ChaC glutathione specific gamma-glutamylcyclotransferase 1 (CHAC1) was upregulated, and H4 clustered histone 3 (H4C3) was downregulated in both HIV-1 and HIV-2 transduced cells. Tables S2 and S3 include all detected transcripts at the 2 h time-point following transduction. We also compared data from HIV-2 transduced cells to those of HIV-1 (Figure 3 and Table S5).

Rather surprisingly, most of the upregulated genes were the same as in the analysis comparing HIV-2 to mock transduced cells, with the exception of the S100A2, DCN and the EIF3CL. More noticeable was the difference of expression in the case of the downregulated protein-coding genes. Compared to HIV-1, the down-regulation of a-kinase anchoring protein 9 (AKAP9), interferon gamma inducible protein 30 (IFI30), centromere protein E (CENPE), oviductal glycoprotein 1 (OVGP1), sosondowah ankyrin repeat domain family member D (SOWAHD), and TNF receptor superfamily member 13C (TNFRSF13C) was more prominent in HIV-2 transduced cells. This clearly indicates a divergent pattern of host genome regulation upon entry between the two viruses. These results were further evaluated by GO term enrichment analysis.

In contrast to the HIV-1, little difference could be observed between the top upregulated protein-coding transcripts in HIV-2 transduced cells between 0 and 2 h time-points. The proteoglycan decorin and S100A6 were uniquely high in the 0 h time-point compared to 2 h post-transduction. Differences were more noticeable between the downregulated protein-coding transcripts, in which MAN1A2, EMP3, SDSL, SKAP1, PHLDA3, and ASS1 were more downregulated at 0 h post HIV-2 transduction

**Figure 4:**
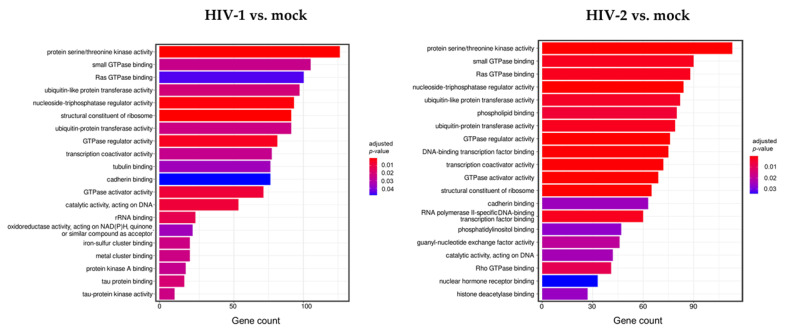
Gene ontology (GO) analysis of differentially expressed genes in HIV-1 (**left** panel) and HIV-2 (**right** panel) transduced HEK-293T cells at 2 h time-point. Figure shows the classification of top 20 genes according to significant enrichment terms Colour intensity corresponds to the significance of each term. Counts represent the number of differentially expressed genes associated with the listed gene ontology ID.

To better understand the biological role of the detected transcripts, gene ontology (GO) enrichment analysis was performed with the most significant, differentially expressed (*p* > 0.05, log_2_FC > 0.58), protein-coding genes at the 2 h time point from both HIV-1 and HIV-2 transduced cells, respectively, compared to mock transduced cells (Figure 4).

Analysis of the data revealed multiple similar biological connections between HIV-1 and HIV-2-influenced genes. Functional association showed that proteins encoded by the detected transcripts were involved in protein serine/threonine kinase activity, ribosome structure, GTPase activity, ubiquitin-like protein transferase, and nucleoside triphosphatase regulator activity. Among the biological pathways of HIV-1 induced genes were rRNA and tau protein binding, tau-protein kinase activity, and catalytic activity on DNA. Proteins coded by HIV-2-influenced genes were found to be involved in Rho GTPase binding, DNA-binding transcription factor binding, transcription coactivator activity, and RNA polymerase II-specific DNA-binding transcription factor binding. A full list of the enriched GO terms can be found in Tables S6 and S7.

### 3.2. Early Proteomic Changes upon HIV-1 and HIV-2 Transduction

**Figure 5:**
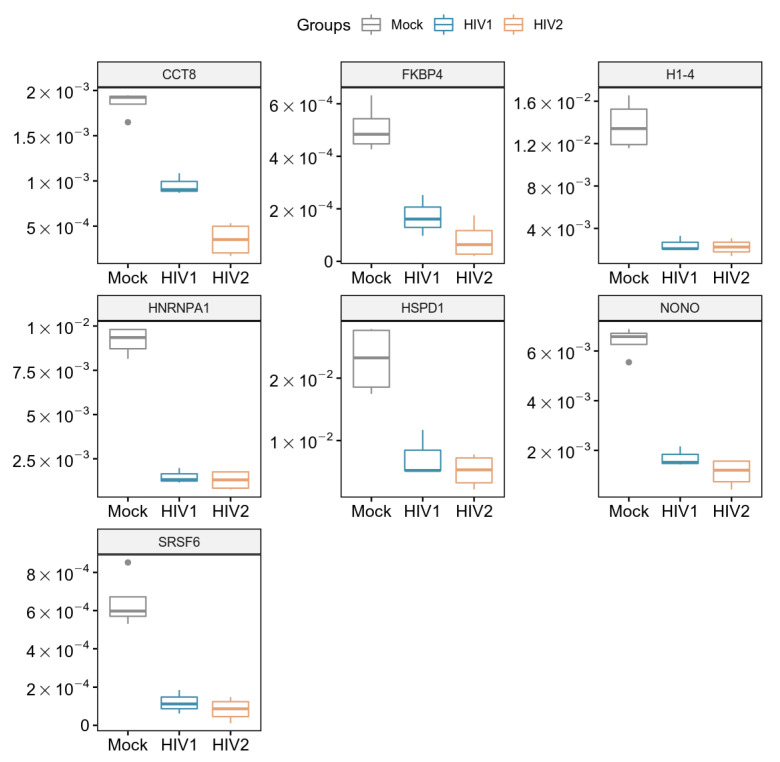
Downregulated proteins at 2 h time-point. Boxplot of the downregulated proteins showing statistically significant changes as a result of HIV-1 and 2 transduction. *p*-value < 0.05. CCT8 and FKBP4 were only significant in HIV-2 transduced cells.

Host cell proteomic analysis revealed over 1000 proteins from transduced samples (Table S8), among which seven were found to be downregulated in a statistically significant manner compared to mock transduction after false discovery rate analysis. Five proteins were downregulated in HIV-1 transduced and two additional proteins in HIV-2 transduced cells compared to the mock transduced cells (Figure 5). Both HIV-1 and -2 downregulated the cellular level of the heterogeneous nuclear ribonucleoprotein A1 (hnRNPA1), non-POU domain containing octamer binding protein (NONO), histone H1.4 (H1-4), mitochondrial 60kDa heat shock protein (HSPD1), and serine/arginine-rich splicing factor 6 (SRSF6). Importantly, FK506-binding protein (FKBP4) and T-complex protein 1 subunit theta (CCT8) were only significantly downregulated in cells transduced with HIV-2 as compared to HIV-1.

Next, the interaction network and biological process enrichment analyses of the seven significantly downregulated proteins were carried out using Cytoscape 3.8.1 [32]. For the physical interaction network, the integrated STRING database with a confidence level of 0.7–100 first shell interactors was queried, and for the gene ontology enrichment Cytoscape’s ClueGO v2.5.7. plugin was used [33].

**Figure 6:**
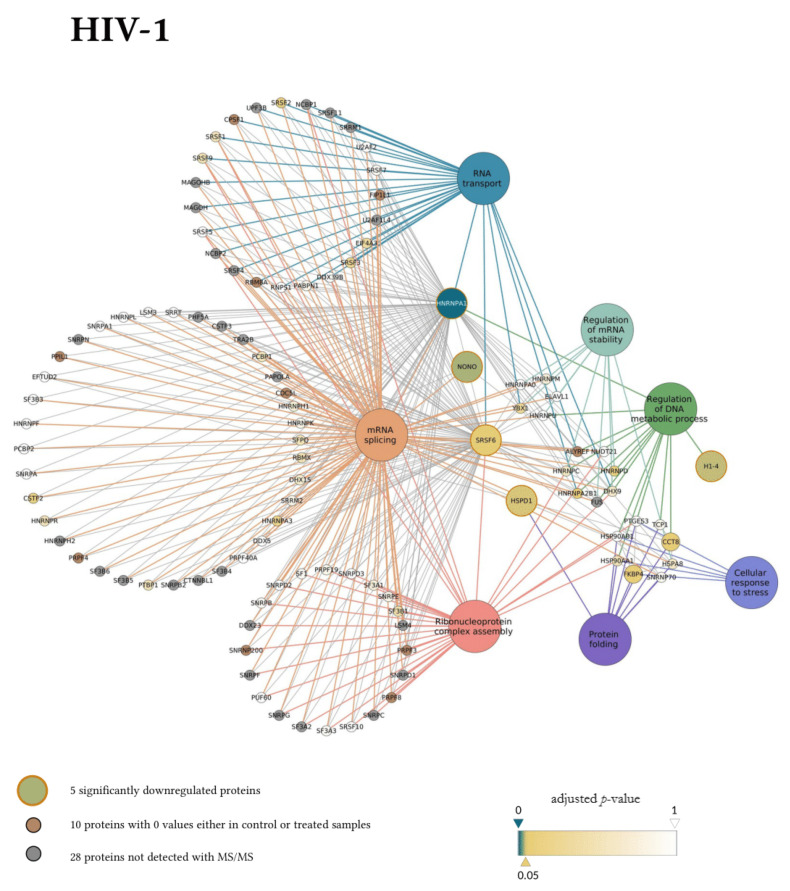

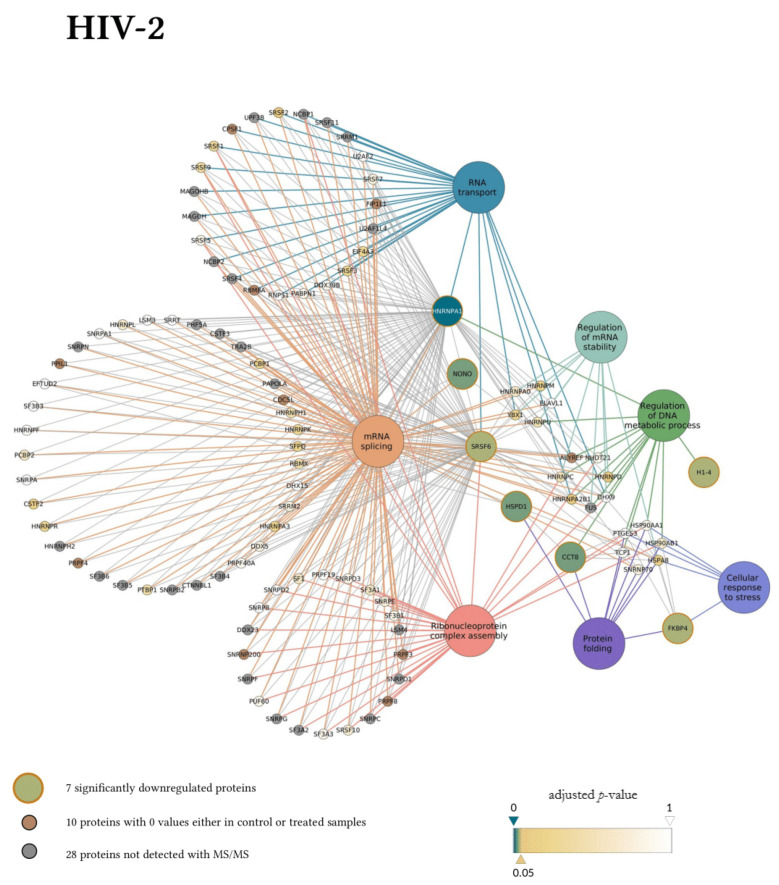
Protein Interaction network of proteins downregulated by HIV-1 (**top** panel) and 2 (**bottom** panel). Downregulated proteins are represented by medium-sized circles. Associated biological process GO term pathways are indicated by the large circles. Smaller circles indicate the interacting proteins, colour and intensity denote the significance of change in their regulation as compared to control (α = 0.05; color scale: *p* = 0—marine blue, *p* = 0.05—gold, *p* = 1—white). Physical protein-protein interactions among the members of the enriched, extended network are not shown.

In the gene ontology analysis of the enriched network that comprised 107 proteins altogether, functional clusters of GO terms were first generated. ClueGO relies on term similarity to define functional groups of multiple terms. In our analysis, initial group size was set to three terms (two being the default value), and the percentage for group merge was left unchanged at the default value of 50%. Further, 33 of the 72 resulting GO terms were grouped into six functional clusters, the rest of them, 39 in total, did not reach the group merge threshold. However, among these was the GO term mRNA splicing (GO:00000398) including 97 proteins in the enriched network (Table S9). Due to the redundancy of GO terms in the clusters, we selected seven biological processes that were the most representative and significant according to their *p*-values corrected with the Bonferroni step-down method (*p* < 0.05). Altogether, 105 proteins were covered by these biological processes in the interaction network (Figure 6). Moreover, 77 of these 105 proteins were also detected by MS/MS, indicating a wider interaction network centred around the significantly downregulated proteins. However, only 67 of these 77 proteins had corresponding quantitative data of sufficient quality to enable statistical analysis, as they could not be quantified in some of the replicate measurements.

**Figure 7:**
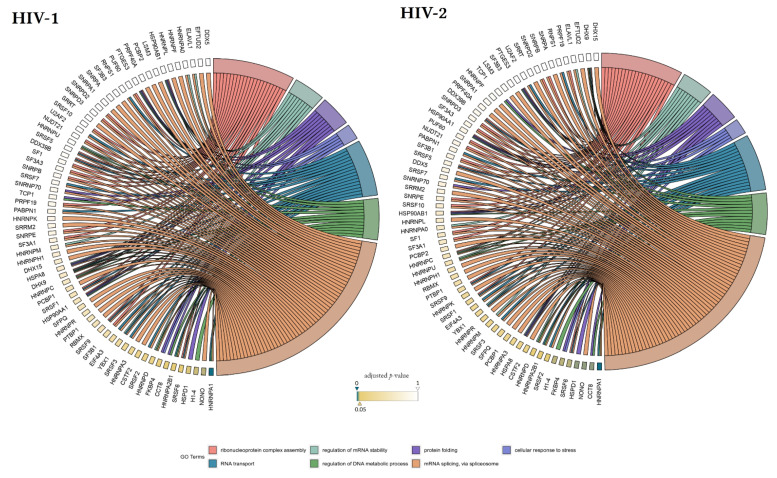
Protein association analysis. Associations between the 7 selected GO terms (ribonucleoprotein complex assembly—GO:0022618, RNA transport—GO:0050658, regulation of mRNA stability—GO:0043488, regulation of DNA metabolic process—GO:0051052, protein folding—GO:0006457, mRNA splicing via spliceosome—GO:0000398, and cellular response to stress—GO:0033554), and proteins from the enriched interaction network also detected by MS/MS. Colour intensities of the rectangles reflect the significance of change in the corresponding protein levels relative to control (α = 0.05; color scale: p = 0—marine blue, p=0.05—gold, p = 1—white). The chords plots were generated with the GOplot R package [38].

Our matching terms, including ribonucleoprotein complex assembly (GO:0022618), RNA transport (GO:0050658), regulation of mRNA stability (GO:0043488), regulation of DNA metabolic process (GO:0051052), protein folding (GO:0006457), mRNA splicing via spliceosome (GO:0000398), and cellular response to stress (GO:0033554), were visualized with the corresponding proteins in Figure 7. Our analyses show that the seven downregulated proteins associate with wide networks of proteins, key members of which are mostly involved in the cellular defence against invading pathogens [34,35,36,37].

## 4. Discussion

Lentivirus-based vectors derived from HIV-1 and -2 are widely used tools in research and may also be utilized in clinical settings. For their safe application, it is of the utmost importance to delineate the changes induced by these virions in the host. HIV-1 and -2 share many similarities, such as their method of transmission and pathogenesis. However, there is a striking difference in their replication dynamics and clinical course of infection. Infection with HIV-2 is characterized by an acute “surge” in viral production right after the infection, followed by a prolonged latency stage [39], lower viral replication [40], and a slower rate of disease progression to AIDS [17]. Factors involved in this dissimilarity in replication dynamics, in contrast to HIV-1, as well as the mechanism of preference for a prolonged latency period of HIV-2 infection remain largely unknown.

While there are adequate proteo-transcriptomic data on host cell changes following HIV-1 infection, there is a contrasting lack of data regarding infection with HIV-2. Moreover, studies have not focused on the very early phase of the infection cycle, mainly characterized by fusion and entry [10].

Our choice of HEK cell and pseudotyping with VSV-G in this study stemmed from our interest in examining the cellular changes induced by the very early entry events of the lentiviruses. Pseudotyping with VSV-G allows the lentivrions to transduce a wide range of cells, since it was shown to utilize the low-density lipoprotein (LDL) receptor as a target [41]. Given the noticeable variability in receptor utilization and infectivity of target cells between HIV-1 and -2, we opted to uniformly pseudotype both viruses with VSV-G, in order to bypass receptor-induced proteo-transcriptomic changes. Previous studies have already reported on Env mediated signaling pathways in target immune cells [42,43]. 

Moreover, HEK-293T cells are widely utilized for lentiviral production due to the expression of the large-T antigen, in addition to the lack of restriction factors and antiviral immune response, hence facilitating superior transfection efficiency and thereafter robust vector productivity [44]. Following production of the desired pseudo-lentivirions, these can then be used to transduce target cells-lines that vary depending on the clinical condition to be corrected.

Pseudotyping with VSV-G and utilizing HEK-293T cells in our experiment therefore enabled us to compare differentially induced genes and affected proteins by HIV-1 and -2 lentiviral-based pseudovirions, untethered from interference by Env/receptor interaction signaling, and the inherent complication of the status of immune cells.

It is also worth noting that some epithelial cells may indeed serve as targets for HIV. Human foreskin epithelial cells were shown to express HIV co-receptors and play an important role in transferring the virions to target immune cells [45]. Others have also documented that lung epithelial cells may also be targeted by HIV-1 and serve as reservoirs during the course of latency [46]. Additionally, epithelial tissues in the tonsils and cervix were also shown to be initially targeted by HIV through cell surface proteins, such as galactosylceramide and heparan sulfate proteoglycans [47].

In our study, RNA-seq analysis was used to determine the changes in cellular gene expression during the very early steps of viral transduction. Our results showed that 4551 genes were differentially regulated by HIV-1 and 3422 by HIV-2, after just 2 h post-transduction.

The difference in differential gene expression between the top 10 most up- and downregulated genes between HIV-1 and HIV-2 transduced cells was indeed significant. Differentially regulated genes by HIV-1 were much more diverse compared to HIV-2, involving chaperones (SACS), DNA repair proteins (TEX15, BRCA2), and transporters (SLC711A). The up-regulation of the cysteine/glutamate antiporter SLC711A by HIV-1 was an interesting finding, as glutamate export from the cell by SLC117A was shown to be increased by the Tat protein of HIV-1, leading to the cellular depletion of glutamate, which results in oxidative stress and exo-cytotoxicity in microglia cells [48]. The glutamate/cysteine level also has an important role in the differentiation of inflammatory dendritic cells (DC). Disturbing the transport could lead to an impaired DC number, and thus inefficient type 1 helper T cell (Th1) activation during HIV infection [49].

The top 10 differentially expressed protein coding genes by HIV-2 were mostly related to extra and intracellular matrix proteins, such as collagen type I alpha 2 chain (COL1A2), type VI alpha 3 chain (COL6A3), and type III alpha chain 1 (COL3A1), keratin 14 (KRT14), 7 (KRT7), and 5 (KRT5). The regulation of collagens has been shown to be altered by the HIV-1 Tat protein during the later stages of infection, along with other extracellular matrix proteins, such as fibronectin and laminin [50]. Moreover, in neuroblastoma cells, extracellular Tat was found to compete with type I collagens of the extracellular matrix, and thus, dysregulating neuronal differentiation [51]. Similarly, the cellular level of cytokeratins can be altered by HIV and other viruses as well, such as human papillomavirus (HPV) [52,53]. However, as far as we know, there is no specific interaction between the proteins of HIV and collagens or cytokeratins. Our finding indicates that only HIV-2 induced the up-regulation of extracellular matrix proteins in a high amount during the very early phase of its life cycle. Dynamic changes in the integrity and composition of the extracellular matrix contributes to the successful immune response against pathogens and communication between the infected and effector cells. Dysregulation of these processes severely inhibits the ability of immune cells to effectively act against infection [54].

Moreover, there is little to no derivation from the top up- and downregulated genes from 0 h and 2 h time points in the case of HIV-2. This might indicate that HIV-2 expresses its pathogenic effect on the cells much later than HIV-1. Furthermore, there is little information about the effect of the VSV.G protein on the cells, especially during early time points. Therefore, the effect that we observed during the 0 h mark, induced by both HIVs, might be the result of variable concentrations of VSV.G. As stated in the methods, the amount of virus was determined by its RT activity, not by the quantity of virions.

Thrombospondin-1, an extracellular matrix glycoprotein, was also highly upregulated by HIV-2. THBS-1 was shown to inhibit extracellular Tat induced HIV-1 LTR transactivation and cell proliferation, along with the ability to bind the gp120 of HIV-1 envelope glycoprotein, subsequently inhibiting viral entry [55,56].

Further analysis of the Gene Ontology database (GO) revealed significant differences in the molecular functions of the affected genes. Both viruses changed the expression profile of genes functionally related to protein processing, protein serine/threonine kinase activity, nucleoside-triphosphatase regulator activity, structural constituent of ribosome, GTPase regulator activity, and GTPase activator activity. We hypothesized that the changes incurred by the above-mentioned genes were mostly a result of cellular response to transduction and viral entry, as proteins with serine/threonine kinase, GTPase activation activity, and GPTase regulation activity are usually associated with signalling, transport, and proliferation. However, some protein kinases are indeed involved in the life cycle of certain viruses, such as HIV, HPV, Ebola, and Influenza A, since the phosphorylation of the viral proteins was found to be necessary for their functionality [57,58,59]. Moreover, small GTPase Rab proteins are utilized by HIV during its later stages of replication for the purpose of trafficking and the maturation of the viral proteins [60].

Changes identified in HIV-1 transduced cells were observed in genes coding for proteins with rRNA binding, tau protein binding, tau-protein kinase activity, iron-sulfur cluster binding, and metal cluster binding.

On the other hand, proteins encoded by HIV-2 affected transcripts were related to Rho GTPase binding, phosphatidylinositol binding, DNA-binding transcription factor binding, guanyl-nucleotide exchange factor activity, and RNA polymerase II-specific DNA binding transcription factor binding. Both viruses altered the expression of proteins with a role in the ubiquitination activity, a post translational modification which is involved in the regulation and degradation of several host proteins. Moreover, this process is also involved in the antiviral response, through regulating the innate RIG-like and Toll like receptor signalling [61]. Several HIV accessory proteins were found to hijack the host cell ubiquitin system in order to initiate degradation of cellular restriction factors and other targets, such as the targeting of SAM domain and HD domain-containing protein 1 (SAMHD1) by HIV-2 Vpx, apolipoprotein B editing enzyme catalytic polypeptide-like 3G (APOBEC3G) by Vif, and Tetherin by HIV-1 Vpu [62,63].

Alongside determining the early transcriptome changes induced by HIV based lentiviral vectors, we also conducted proteomic analysis that revealed that in the first 2 h of transduction, both viruses resulted in a significant down-regulation of hnRNPA1, NONO, H1-4, HSPD1, and SRSF6 to similar levels, although the down-regulation of CCT8 was more evident following transduction with HIV-2 compared to HIV-1 and the time-matched mock transduction.

hnRNPA1 is a member of a complex family of ribonucleoproteins, and plays a role in the transcription, stability, and transport of newly synthetized cellular mRNAs. It was shown to act as a splice inhibitor of HIV-1 *tat* mRNA following expression from the integrated proviral genome, binding to specific sites on the newly synthesized transcript [64]. This is indeed beneficial for the virus, preventing the overexpression of Tat, which is highly apoptotic. Additionally, through Rev stimulation, it aids in the viral mRNA transport from the nucleus to the cytoplasm [65]. In the context of viral infection, hnRNPA1 plays a major functional and regulatory role, and while it was found to enhance some viral infections, such as sindbis virus, enteroviruses, and rhinovirus, it counteracted Human T cell lymphotropic virus (HTLV-1) and hepatitis C virus (HCV) infections [35].

Interestingly, previous studies have shown that HIV-1 enhances the expression and cytoplasmic relocalization of hnRNPA1 during the late-phase of infection, in order to facilitate adequate viral protein expression before budding [66]. We, however, found that in the immediate early stage of transduction, levels of hnRNPA1 were significantly downregulated by both HIV-1 and 2, which may perhaps indicate a cellular defence mechanism limiting viral replication.

NONO is a nuclear protein involved in RNA splicing and transcriptional regulation and was also found to play a role during the course of HIV infection, as previous studies have documented its association with the reverse transcription and preintegration complexes [67,68]. In Jurkat cells, the overexpression of NONO resulted in a down-regulation of HIV-1 infectivity, through negatively affecting reverse transcription and proviral genome expression. Although the mechanism remains unclear, it is thought that this down-regulation of reverse transcription is either accomplished through direct interaction with the reverse transcription complex, mediated by its DNA and RNA binding motifs, or indirectly through interaction with other components involved in the process [68]. Additionally, NONO, as part of the HEXIM1-DNAPK-Paraspeckles components-ribonucleoprotein complex (HDP-RNP), is required for the innate immune response against foreign DNA, through the cGAS-STING-IRF3 pathway [34].

SRSF6 is an alternative splicing regulator that is capable of interaction with the splicing sites of the Tat 3′ss A3 mRNA and may be required for the sufficient activation of splicing [69]. Overexpression of the protein was found to severely impair HIV-1 proviral genome expression by a mechanism that is yet to be defined.

HSPD1 is able to interact with the viral gp41 protein and thus become incorporated in the newly formed virions [70]. Moreover, during the early-phase of the HIV-1 life cycle, HSPD1, as a member of the PIC, binds to HIV-1 integrase and protects it from denaturation [71]. The incorporation of HSPD1 into the virion could ensure the stability and folding of the HIV integrase in preparation for the upcoming infection, especially when the cellular level of heat shock proteins is decreased. Previous studies have also indicated that the level of HSPD1 in the serum correlates with the level of viral load and decreases during anti-retroviral therapy [72]. HSPD1 is apparently utilized not only by HIV, but other viruses, such as hepatitis B and influenza A, to ensure an effective replication as well [37,73].

Scarce information exists in relation to HIV and H-1.4 histone protein, other than its presence at the viral integration site and that it may have a role in the repression of Tat mediated transcription [74].

FKBP4 is an essential mediator of nuclear translocation and was shown to possess an antiviral effect against herpes simplex virus 1 (HSV-1) [36]. Levels of FKBP4 were found to be highly elevated in the brain during HIV generated inflammation and astrocyte activation [75]. Moreover, others have shown that its level was increased following the expression of HIV-1 Tat in Jurkat cells [76].

Lastly, TCP1 chaperon complex member 8 (CCT8) was found to be an important marker for disease progression towards AIDS, showing low expression during slow/non- progression, and increasing correspondingly in AIDS [77]. Moreover, TCP1 is able to interact with the HIV accessory protein Vif. However, the importance of this interaction has not yet been uncovered [78]. How HIV-2 was able to downregulate CCT8 to levels well below those detected in HIV-1 transduced cells is indeed an interesting finding, and whether or not this has an implication in the decreased pathogenicity of HIV-2 warrants further investigation.

A likely mechanism explaining the decrease of the cellular proteins detected in our studied timeframe is proteolytic degradation, either by the viral protease entering the cells within the viral core or by rapid induction of the degradation by cellular proteolytic systems, such as the involvement of proteasomal degradation. In this aspect, it is important to note that hnRNPA1, NONO, FKBP4, and HSPD1 can act as substrates for the HIV protease [79].

STRING and GO term analysis revealed the complex network of cellular processes the aforementioned proteins are involved in, including mRNA splicing, mRNA stability, protein folding, and cellular response to stress. It is also important to note that the initial decrease that we observed in the level of these proteins might be overridden later, as the HIV life cycle advances to later stages. Further experiments are needed to uncover the possible mechanisms responsible for their up-regulation during later events in the life cycle of HIV. While some of the proteins identified in the proteomic analysis were not reflected in the transcriptomic analysis, it is worth noting that transcriptomic changes are not always reflected by protein level, given the substantial role of cellular post-transcriptional and post-translational regulation influencing protein production, and also taking into consideration the timepoint at which the analysis was carried out [80].

In conclusion, analysis of the proteo-transcriptomic data in the first 2 h following transduction of cells with pseudotyped HIV-1 and -2 based virions indicates that the cellular response in the immediate early phase of transduction is significantly different between lentiviral vectors based on the two viruses. The results shown in our study hint to the association and implication of individual viral capsid proteins, enzymes, and accessory proteins in inducing these changes. While we have not analyzed these changes in primary target immune cells, we believe that this manuscript provides important information on the pathomaechanistic aspect of transduction with lentivectors. Additionally, to our knowledge, studies of the effects of HIV transduction in the very early times-points are indeed lacking, and more so in the case of HIV-2, and although our lentivirions were pseudotyped with VSV.G to facilitate the transduction of HEK-293T cells, our findings delineate the non-Env mediated cellular changes induced by transduction and may provide an insight into the understudied replication cycle of HIV-2, enriching our knowledge about the use of HIV-based lentiviral vectors as a whole.

## Data Availability

Proteomic data presented in this study are openly available in PRIDE at 10.6019/PXD024243, reference number PXD024243. Transcriptomic data presented in this study are openly available in GEO at GSE167098, reference number 200167098.

## Supplementary Materials

The following are available online at https://www.mdpi.com/article/10.3390/microorganisms9112207/s1, Table S1: Overview of sequencing and alignment data. Table S2: Full list of the detected RNA transcripts from HIV-1 transduced cells compared to mock 2 h after transduction. Table S3: Full list of the detected RNA transcripts from HIV-2 transduced cells compared to mock 2 h after transduction. Table S4: Full list of the detected RNA transcripts from HIV-1 and HIV-2 transduced cells compared to mock and to each other 0 h after transduction. Table S5: Full list of the detected RNA transcripts from HIV-2 transduced cells compared to HIV-1 transduced cells 2 h after transduction. Table S6: List of GO enrichment terms of RNA transcripts detected from HIV-1 transduced cells compared to mock-transduced cells 2 h after transduction. Table S7: List of GO enrichment terms of RNA transcripts detected from HIV-2 transduced cells compared to mock-transduced cells 2 h after transduction. Table S8: Full list of the detected proteins after 2 h from both HIV-1 and HIV-2 transduced samples, respectively. Table S9: List of the 77 proteins detected from both HIV-1 and HIV-2 transduced 2-h samples and from the interaction analysis.
